# The preoperative neutrophil‐to‐lymphocyte ratio is not a marker of prostate cancer characteristics but is an independent predictor of biochemical recurrence in patients receiving radical prostatectomy

**DOI:** 10.1002/cam4.1984

**Published:** 2019-01-28

**Authors:** Zhi Cao, Jin Ji, Chao Zhang, Fubo Wang, Huan Xu, Yongwei Yu, Yinghao Sun

**Affiliations:** ^1^ Department of Urology Changhai Hospital Navy Medical University Shanghai P. R. China; ^2^ Department of Pathology Changhai Hospital Navy Medical University Shanghai P. R. China

**Keywords:** biochemical recurrence, lymphocyte–neutrophil ratio, prostate cancer, radical prostatectomy

## Abstract

The neutrophil‐to‐lymphocyte ratio (NLR) has been reported to be a prognostic marker in prostate cancer. In this study, we assessed the association between preoperative NLR and the clinicopathological characteristics, biomolecular features and prognosis of patients with localized prostate cancer treated with radical prostatectomy. A total of 994 subjects were retrospectively enrolled, and the histological specimens of 210 patients were retrieved for constructing a tissue microarray. Immunohistochemistry was then performed to assess the expression of AR, ERG, PTEN, p‐AKT, Bcl‐2, Beclin‐1, Ki‐67, CD3, CD4, CD8, IFN‐γ and TNF‐α. No significant differences in the NLR distributions among clinicopathological variables were observed (*P* > 0.05) when the original NLR data were utilized. When we dichotomized the NLR value into the high‐NLR group (NLR ≥ 2) and low‐NLR group (NLR < 2), we found that the patients in the high‐NLR group had more prostate capsule invasion (*P* = 0.047). Additionally, no significant correlation was found between the NLR and infiltrating CD3^+^ cells, the CD4/CD8 ratio, AR, ERG, PTEN, p‐AKT, Bcl‐2, Beclin‐1, Ki‐67, IFN‐γ or TNF‐α (*P* > 0.05). When we analyzed the data of patients without postoperative adjuvant hormone therapy or radiotherapy, univariate and multivariate survival analysis indicated that a high NLR was a predictor of better BCR‐free survival (*P* < 0.05). When analyzing the entire cohort, univariate survival analysis showed that the high‐NLR group had significantly poorer overall survival (*P* < 0.05). In conclusion, NLR cannot reflect prostate cancer characteristics or the local immune microenvironment, but a high NLR can serve as an independent predictor of better BCR.

## 1 INTRODUCTION

Prostate cancer is the most common malignant tumor and a leading cause of cancer‐related death in men worldwide.^1^ The prostate cancer burden in Chinese men has continuously increased due to increased screening based on blood prostate specific antigen (PSA) test, altered lifestyles and the aging of society.^2^ Radical prostatectomy (RP) is the first choice for the management of localized prostate cancer (LPCa). Unfortunately, a large proportion of LPCa patients progress to recurrence after RP.^3^ Therefore, reliable, easily accessible and inexpensive predictors for individualized risk assessment and clinical decision‐making are urgently needed.

The neutrophil‐to‐lymphocyte ratio (NLR), which combines peripheral blood neutrophil and lymphocyte counts, has been proposed as an indicator of the host inflammatory status and general immune response to various stress stimuli.^3^, ^4^ Previous studies have indicated the correlation between NLR and the prognoses of patients with various malignant tumors.^5^, ^6^, ^7^, ^8^ For castration‐resistant prostate cancer (CRPC), a high NLR is reported to be correlated with poor prognosis.^9^, ^10^ Recent reports have also found that an elevated NLR is associated with early biochemical recurrence (BCR) in patients treated with RP.^3^, ^11^, ^12^ Another study showed that LPCa patients with high NLRs experience worse prognoses in terms of progression‐free, metastasis‐free, and overall survival after radiotherapy.^13^ However, the evidence of using the NLR to predict the prognosis of prostate cancer is limited. Few studies have explored correlations between pretreatment NLR and indexes that can directly represent prostate cancer characteristics and local microenvironments.

In this study, we examined the association between preoperative NLR and the pathological features and representative molecules of biological function and the local microenvironment, such as infiltrating CD3^+^ cells, the CD4/CD8 ratio, AR, ERG, PTEN, p‐AKT, Bcl‐2, Beclin‐1, Ki‐67, IFN‐γ and TNF‐α in LPCa, and we determined whether pretreatment NLR could predict the prognosis of LPCa patients receiving RP.

## 2 MATERIALS AND METHODS

This retrospective study was carried out at the Department of Urology in Changhai Hospital and got the approval of the medical ethics review committee of Changhai Hospital.

### 2.1 Patient selection and follow‐up

Cases listed in the prostate cancer database of our department were screened to recruit patients who underwent RP and had a record of preoperatively measured blood cell counts from the peripheral blood. To ensure that the preoperative blood cell counts were not affected by other confounding factors, patients who met the following criteria were excluded: a blood routine examination within 2 weeks after any surgical intervention, including prostate biopsy; acute infection or antibiotic or chronic steroid medication use; a history of concurrent malignant tumors in other organs or systemic inflammatory disease; blood transfusion within 12 weeks; or neoadjuvant therapy for prostate cancer. The preoperative clinical characteristics and postoperative pathological features were acquired from our database and medical records. The NLR was calculated as follows: NLR = neutrophil count/lymphocyte count. Additionally, only blood routine test before prostate biopsy or radical prostatectomy was collected, and the mean NLR was taken as the representative value if multiple blood routine tests were performed. The follow‐up data were acquired by reviewing our database and contacting patients or their family members. BCR was defined as at least two consecutive PSA test ≥0.2 ng/mL according to the guidelines of the American Urological Association.^14^ Additionally, postoperative adjuvant hormonal therapy or radiotherapy was administered to those patients who met the criteria according to the guidelines for prostate cancer established by the Chinese Urological Association.

An online tool was applied to calculate the sample size, the website is http://powerandsamplesize.com/Calculators/Test-Time-To-Event-Data/Cox-PH-2-Sided-Equality. The primary study outcome was set as the difference of BCR between high‐ and low‐NLR groups. And the study was designed to have a statistical power of 0.8 to detect a difference between groups, on the basis of 5% type I error rate, 30% probability of BCR, equal distribution of patients between groups, a previously reported hazard ratio of 1.5 and a two‐sided alpha value of 0.05.^3^, ^11^ These calculations indicate that 636 patients need to be recruited, and the number was increased to 994 after considering the retrospective character of the study.

### 2.2 Tissue microarray construction

Available histological specimens of the above enrolled prostate cancer patients with complete follow‐up data were retrieved from the archives of the Department of Pathology. The histological sections were reviewed by our pathology team to identify the most representative areas of each sample for construction of the tissue microarray (TMA), both tumor tissue and para‐cancerous tissue were identified. Two tissue cores (1.5 mm in diameter) from each case were selected and embedded in the recipient block, and serial 3‐mm sections were cut for further research.

### 2.3 Immunohistochemistry and evaluation

Immunohistochemistry was performed to assess the expression of AR (rabbit polyclonal, 22089‐1‐AP, Proteintech), ERG (rabbit monoclonal, ab133264, Abcam), PTEN (rabbit polyclonal, 22034‐1‐Ig, Proteintech), p‐AKT (mouse monoclonal, 66644‐1‐Ig, Proteintech), Beclin‐1 (rabbit polyclonal, 11306‐1‐Ig, Proteintech), Bcl‐2 (rabbit polyclonal, 12789‐1‐AP, Proteintech), Ki‐67 (rabbit polyclonal, 27309‐1‐AP, Proteintech), CD3 (rabbit polyclonal, 17617‐1‐AP, Proteintech), CD4 (rabbit polyclonal, 19068‐1‐AP, Proteintech), CD8 (rabbit polyclonal, ab4055, Abcam), IFN‐γ (rabbit polyclonal, 18013‐1‐AP, Proteintech), and TNF‐α (rabbit polyclonal, 17590‐1‐AP, Proteintech).

**Figure 1:**
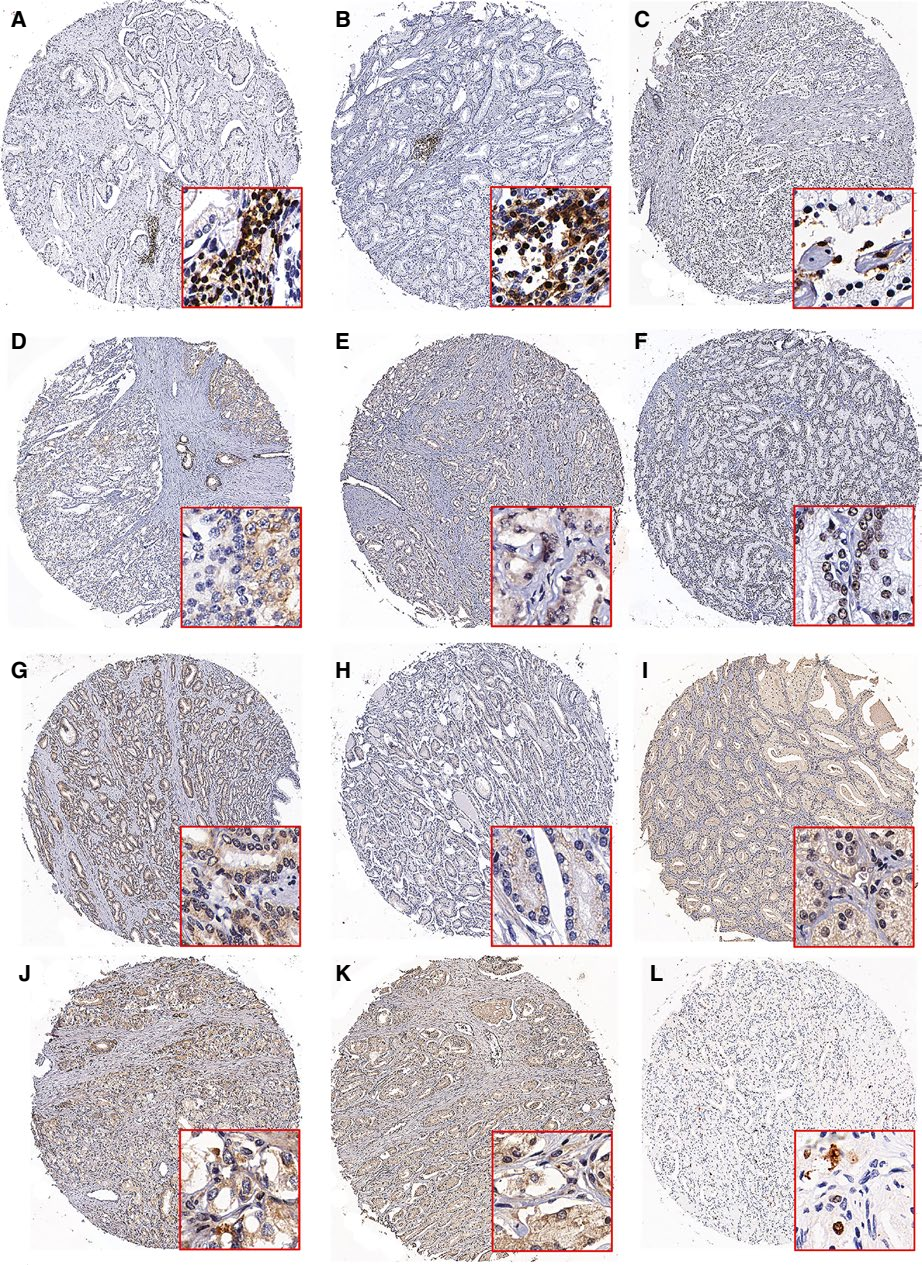
Representative specimens demonstrating the expression of CD3 (A), CD4 (B), CD8C (C), TNF‐α (D), IFN‐γ (E), AR (F), ERG (G), p‐Akt (H), PTEN (I), Bcl‐2 (J), Beclin‐1 (K) and Ki‐67 (L) in prostate cancers by IHC

The expression of AR, ERG, PTEN, p‐AKT, Bcl‐2, Beclin‐1, IFN‐γ, and TNF‐α was quantified using the Pannoramic viewer and Quant center image analysis software (3D HISTECH, Hungary) to calculate the H‐score. The H‐score was calculated as follows: percent of weak staining (scale: 0‐100)*1 + percent of moderate staining (scale: 0‐100)*2 + percent of strong staining (scale: 0‐100)*3. For Ki‐67, the results were represented as the ratio of positive cells to total cancer cells. The density of tissue infiltrating lymphocytes (CD3^+^, CD4^+^ and CD8^+^) was calculated as the counts of CD3‐, CD4‐ and CD8‐positive cells per mm^2^. Representative staining specimens are shown in Figure 1.

### 2.4 Statistical analysis

Statistical analysis was conducted by applying SPSS 13.0 (SPSS Inc., Chicago, IL, USA). The independent *t* test (two sets of independent variables) and one‐way ANOVA (three or more sets of independent variables) were performed to calculate the difference between continuous variables, while Chi‐square test was performed to examine categorical variables. Spearman correlation analysis was applied to explore the correlation between NLR and infiltrating CD3^+^ cells, the CD4/CD8 ratio, AR, ERG, PTEN, p‐AKT, Bcl‐2, Beclin‐1, Ki‐67, IFN‐γ and TNF‐α. The X‐tile software (Yale University, USA) was used to calculate the optimal NLR cutoff value. The log‐rank test and Cox proportional hazard regression analysis were utilized to assess the association between NLR and survival. *P* < 0.05 was taken as statistically significant.

## 3 RESULTS

A total of 994 patients were eventually enrolled in the study, and the histological specimens of 210 patients with complete follow‐up data were applied to construct the TMA. A total of 188 patients received postoperative adjuvant hormone therapy or remedial radiotherapy due to a positive surgical margin, positive lymph node invasion, positive seminal vesicle invasion, high pT stage or an unsatisfactory decline of PSA. The remaining 806 patients were termed prostate cancer patients with successful radical prostatectomy (PCASRP). The demographic and clinicopathological characteristics are detailed in Table 1.

**Table 1:** Clinical and pathological features of the enrolled patients

Variables	Prostate cancer (All, n = 994)	Prostate cancer (TMA, n = 210)
Age (years)	67.1 ± 7.1	65.7 ± 10.6
BMI	24.24 ± 3.05	24.34 ± 2.75
Preoperation PSA (ng/mL)	24.90 ± 53.5 (n = 963)	40.38 ± 102.96 (n = 208)
Preoperation f/tPSA (ng/mL)	0.12 ± 0.08 (n = 613)	0.11 ± 0.09 (n = 140)
pT category (AJCC 2002)		
pT2	663 (66.7%)	121 (57.6%)
pT3	275 (27.6%)	82 (39.0%)
PT4	24 (2.4%)	7 (3.4%)
NA	32 (3.2%)	
Gleason grade		
≤3 + 3	180 (18.1%)	11 (5.2%)
3 + 4	331 (33.3%)	66 (31.4%)
4 + 3	171 (17.2%)	37 (17.6%)
≥4 + 4	262 (26.4%)	96 (45.8%)
NA	50 (5%)	
pN category		
pN0	724 (72.9%)	130 (61.9%)
pN+	58 (5.8%)	43 (20.5%)
NA	212 (21.3%)	37 (17.6%)
Prostate capsule invasion		
Negative	726 (73.0%)	128 (61.0%)
Positive	250 (25.2%)	82 (39.0%)
NA	18 (1.8%)	
Seminal vesicle invasion		
Negative	820 (82.5%)	157 (74.8%)
Positive	155 (15.6%)	53 (25.2%)
NA	19 (1.9%)	
Surgical margin		
Negative	631 (63.5%)	114 (54.3%)
Positive	344 (34.6%)	96 (45.7%)
NA	19 (1.9%)	
Nerve invasion		
Negative	653 (65.7%)	113 (53.8%)
Positive	322 (32.4%)	97 (46.2%)
NA	19 (1.9%)	
Follow‐up time (Days)	1295.1 ± 631.6	681.2 ± 617.5
Number of biochemical recurrence	346	128
Number of death	36	8

TMA, tissue microarray; BMI, body mass index; PSA, prostate specific antigen; NA, not available.

To determine the optimal NLR cutoff value, the X‐tile software was applied to evaluate the efficacy of NLR as a predictor for BCR of PCASRP, which indicated that an NLR value of 2 showed the best distinction. Therefore, the NLR cutoff value for BCR was set at 2. The neutrophil counts of the low‐NLR group (NLR < 2) were significantly lower than those of the high‐NLR group (NLR ≥ 2) (3.00 ± 0.89 vs 4.44 ± 1.84, *P* < 0.001), while the lymphocyte counts of the low‐NLR group were significantly higher than those of the high‐NLR group (2.13 ± 0.64 vs 1.48 ± 0.48, *P* < 0.001). When the original NLR data were utilized for statistical analysis, no significant differences in NLR distribution among the clinicopathological parameters of prostate cancer were observed (*P* > 0.05, Table 2). When we dichotomized the NLR value into the low‐NLR group and the high‐NLR group, we found that patients in the high‐NLR group had more prostate capsule invasion than patients in the low‐NLR group (*P* = 0.047, Table 2). No significant association was found between NLR and infiltrating CD3^+^ cells (n = 206, *r* = −0.0656, *P* = 0.352), the CD4/CD8 ratio (n = 205, *r* = 0.073, *P* = 0.297), IFN‐γ (n = 206, *r* = −0.016, *P* = 0.821), TNF‐α (n = 206, *r* = −0.018, *P* = 0.802), Ki‐67 (n = 205, *r* = −0.091, *P* = 0.197), ERG (n = 198, *r* = −0.096, *P* = 0.178), AR (n = 201, *r* = −0.024, *P* = 0.731), p‐Akt (n = 201, *r* = −0.013, *P* = 0.856), Bcl‐2 (n = 202, *r* = −0.081, *P* = 0.252), Beclin‐1 (n = 194, *r* = 0.001, *P* = 0.999) or PTEN (n = 197, *r* = 0.02, *P* = 0.779).

**Table 2:** Association between neutrophil‐to‐lymphocyte ratio (NLR) and pathological variables

Variable	No. of patients (n = 994)	NLR (Mean ± SD)	*P*‐value	Low	High	*P*‐value
pT stage	958 (100%)		0.650			0.115
pT2	660 (68.9%)	2.40 ± 2.35		362 (69.9%)	298 (67.7%)	
pT3	274 (28.6%)	2.29 ± 1.90		148 (28.6%)	126 (28.6%)	
pT4	24 (2.5%)	2.67 ± 1.45		8 (1.5%)	16 (3.7%)	
Gleason grade	940 (100%)		0.312			0.255
≤3 + 3	180 (19.1%)	2.37 ± 2.04		99 (19.6%)	81 (18.6%)	
3 + 4	330 (35.1%)	2.25 ± 1.83		185 (36.6%)	145 (33.4%)	
4 + 3	170 (18.1%)	2.65 ± 3.36		95 (18.8%)	75 (17.2%)	
≥4 + 4	260 (27.7%)	2.38 ± 1.84		126 (25.0%)	134 (30.8%)	
Preoperation PSA level (ng/mL)	958 (100%)		0.111			0.946
PSA≤10	327 (34.1%)	2.21 ± 1.70		178 (34.3%)	149 (33.9%)	
PSA>10	631 (65.9%)	2.45 ± 2.43		341 (65.7%)	290 (66.1%)	
pN stage	778 (100%)		0.268			0.680
pN0	721 (92.7%)	2.39 ± 2.33		395 (92.3%)	326 (93.1%)	
pN+	57 (7.3%)	2.76 ± 3.18		33 (7.7%)	24 (6.9%)	
Prostate capsule invasion	972 (100%)		0.376			0.047
Negative	723 (74.4%)	2.33 ± 2.25		405 (77.0%)	318 (71.3%)	
Positive	249 (25.6%)	2.47 ± 2.04		121 (23.0%)	128 (28.7%)	
Seminal vesicle invasion	971 (100%)		0.800			0.052
Negative	817 (84.1%)	2.38 ± 2.27		453 (86.3%)	364 (81.6%)	
Positive	154 (15.9%)	2.33 ± 1.81		72 (13.7%)	82 (18.4%)	
Surgical margin	971 (100%)		0.772			0.166
Negative	627 (64.6%)	2.38 ± 2.28		247 (58.1%)	280 (62.8%)	
Positive	344 (35.4%)	2.34 ± 2.04		178 (41.9%)	166 (37.2%)	
Nerve invasion	971 (100%)		0.710			0.733
Negative	649 (66.8%)	2.35 ± 2.16		349 (66.3%)	300 (67.4%)	
Positive	322 (33.2%)	2.40 ± 2.28		177 (33.7%)	145 (32.6%)	

PSA, prostate specific antigen.

**Figure 2:**
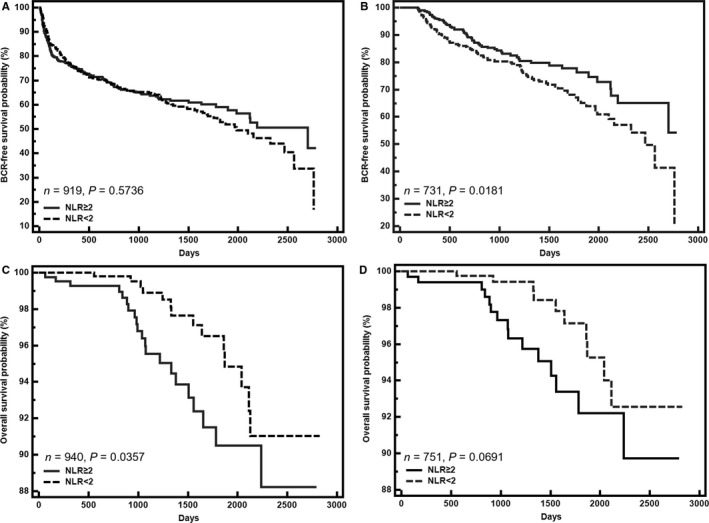
Kaplan–Meier curves for biochemical recurrence (BCR)‐free survival and overall survival according to the neutrophil–lymphocyte ratio in the entire cohort (A and C) and in patients without postoperative adjuvant hormone therapy and radiotherapy (B and D)

Further survival analysis was conducted, and the follow‐up information is presented in Table 1. When we included all patients in the survival analysis, univariate and multivariate survival analysis indicated that NLR was not a predictor of BCR‐free survival (*P* > 0.05, Figure 2A and Table S1). Kaplan‐Meier analysis and univariate Cox proportional hazard regression analysis showed the high‐NLR group had a significantly poorer overall survival rate than the low‐NLR group (*P* = 0.0357, Figure 2C, and *P* = 0.040, Table S1). However, this finding was not validated in the multivariate Cox proportional analysis after adjusting for confounding variables including pT stage, pN stage, Gleason grade and PSA level (*P* = 0.374, Table S1). When we only analyzed the data for PCASRP, Kaplan‐Meier analysis showed that high NLR was a predictor of better BCR‐free survival (*P* = 0.0181, Figure 2B) but not a predictor of overall survival (*P* = 0.0691, Figure 2D). The high‐NLR group also showed significantly better BCR‐free survival (*P* = 0.019) in the univariate Cox proportional analysis, and high NLR was revealed as an independent favorable predictor of BCR in the multivariate Cox proportional analysis (*P* = 0.007, Table S3). Univariate and multivariate Cox proportional analyses confirmed that there was no association between NLR and overall survival (*P* = 0.075 and *P* = 0.395, respectively, Table 3).

**Table 3:** Cox univariate and multivariate analyses of survival according to neutrophil‐to‐lymphocyte ratio (NLR) in patients without postoperative adjuvant hormone therapy or radiotherapy

	BCR‐free time		Overall survival time	
	HR (95%CI)	*P*‐value	HR (95%CI)	*P*‐value
Univariate analysis				
NLR		0.019		0.075
<2	1		1	
≥2	0.680 (0.493‐0.938)		2.068 (0.929‐4.606)	
Multivariate analysis				
NLR		0.007		0.395
<2	1.000		1.000	
≥2	0.600 (0.413‐0.871)		1.466 (0.607‐3.542)	
pT stage		0.002		0.176
Low (pT2)	1		1	
High (pT3‐4)	1.794 (1.230‐2.616)		0.452 (0.143‐1.427)	
pN stage		0.698		0.550
Negative	1		1	
Positive	1.171 (0.528 ‐2.595)		1.900 (0.232‐15.556)	
Gleason grade		<0.001		0.114
Low (<4 + 4)	1		1	
High (≥4 + 4)	3.317 (2.294‐4.797)		2.184 (0.830‐5.750)	
PSA level		0.098		0.376
Low (≤10 ng/mL)	1.000		1.000	
High (>10 ng/mL)	1.425 (0.937‐2.166)		1.552 (0.587‐4.106)	

PSA, prostate specific antigen.

## 4 DISCUSSION

The present study indicated that preoperative NLR was not associated with clinicopathological characteristics (e.g., pT stages, Gleason grades, preoperative PSA levels, pN stages, prostate capsule invasion status, seminal vesicle invasion status, surgical margin status and nerve invasion status), biomolecular features (e.g., AR, ERG, PTEN, p‐AKT, Bcl‐2, Beclin‐1 and Ki‐67) and the local immune microenvironment (e.g., CD3^+^ cells, CD4/CD8 ratio, IFN‐γ and TNF‐α) of LPCa. In addition, high NLR was shown to be an independent favorable predictor of BCR.

The NLR has been proposed as an indicator of general immune response to various stress stimuli and the host inflammatory status. Some researchers hold the view that NLR can, at least to some extent, represent the features and microenvironment of LPCa.^3^, ^11^, ^12^, ^15^ Lee reported that a high NLR (≥2.5) is positively associated with Gleason score, pathological stage and extracapsular extension.^11^ Another study indicated that a high NLR (≥2.36) has an increased risk of high pathological stage and lymph node involvement.^3^ Notably, the two above‐mentioned studies dichotomized NLR and then applied Chi‐square tests to conduct the statistical analyses, and the distribution of patients between the low‐NLR group and high‐NLR group was unequal. Similarly, the present study also found that the high‐NLR group had more prostate capsule invasion when we dichotomized the NLR value into the low‐NLR group and high‐NLR group. However, when the original NLR data were utilized for statistical analysis, the independent *t* tests and one‐way ANOVA did not reveal significant differences in the NLR distributions among pT stages, Gleason grades, preoperative PSA levels, pN stages, prostate capsule invasion status, seminal vesicle invasion status, surgical margin status or nerve invasion status. Interestingly, neither of the two above‐mentioned studies reported these results. One can reasonably doubt that the significant difference in the NLR distribution among the variables might be determined by the different efficiencies of the statistical tests. Furthermore, this study found no significant correlation between NLR and infiltrating CD3 cells, the CD4/CD8 ratio, AR, ERG, PTEN, p‐AKT, Bcl‐2, Beclin‐1, Ki‐67, IFN‐γ and TNF‐α. Given the above evidence, one cannot argue that the NLR reflects the clinicopathological characteristics, molecular features or local immune microenvironment of LPCa.

The most attractive feature of NLR for urologists originates from its prognostic value in prostate cancer.^3^, ^4^, ^9^, ^10^, ^11^ Lee et al showed that a high pretreatment NLR is an independent predictor of worse BCR after RP. A study from China also verified that a high NLR was correlated with a shortened BCR‐free survival time after RP, although the results were not verified by multivariate analysis.^3^ A common phenomenon is that a certain proportion of prostate cancer patients receive postoperative adjuvant hormone therapy or remedial radiotherapy. The above studies did not clarify this issue in the methods or report corresponding results, so hidden bias may exist. Additionally, the unequal distribution of patients between the low‐ and high‐NLR groups may reduce the power of the statistical analysis. Several other studies have focused on CRPC. Nuhn et al analyzed 238 metastatic CRPC patients treated with docetaxel chemotherapy and showed that a low NLR is an independent predictor of longer overall survival.^9^ In another retrospective analysis of 784 CRPC patients treated with prednisone and sunitinib, Sonpavde et al found that a high NLR is an independent poor predictor of overall survival.^16^ Similarly, another study analyzed 108 CRPC patients treated with abiraterone and reported that a low NLR is associated with a better PSA response and prolonged overall survival.^17^

In the present study, we separately analyzed the BCR‐free survival and overall survival of the entire cohort and PCASRP. The results showed that a high NLR is an independent predictor of better BCR‐free survival for PCASRP, while such a correlation was not found in the entire cohort. Additionally, univariate analysis indicated that a high NLR was associated with poor overall survival in the entire cohort, but this correlation was not verified in the multivariate analysis. A similar trend was observed in PCASRP (Figure 2D), but no significant correlation between the NLR and overall survival was calculated in PCASRP. The above evidence indicates that doctors should be cautious of whether patients receive postoperative adjuvant therapy when applying NLR to predict the prognosis of prostate cancer. It is worthy to note that only 36 (3.62%) patients died during the follow‐up. Utilizing these data to evaluate the value of NLR in predicting overall survival seems to be not solid. We observed an emerging association of high NLR and poor overall survival in the PCASRP group, the *P*‐value of which is close to 0.05. So we will continuously follow up patients of the cohort to clarify this issue in the future.

The results of the present study seem to contradict those of previous reports and are hard to explain. Previous studies have indicated that malignant tumors can produce bioactive molecules to increase granulopoiesis and decrease lymphopoiesis.^18^, ^19^, ^20^, ^21^ The decreased lymphocyte counts are correlated with an immunosuppressive status in many cancers, which then decrease the efficacy of the immune system in resisting or eradicating the formation and progression of malignant tumors.^22^, ^23^ Additionally, inflammatory cells can secrete a series of biomolecules, such as TNF, interleukin‐1, and interleukin‐6, which stimulate proliferative signaling, induce resistance to apoptosis, maintain DNA mutations, and promote angiogenesis, invasion, and metastasis.^22^, ^24^ The above evidence reveals that a high NLR reflects both a heightened neutrophil‐dependent inflammatory reaction and a lesser lymphocyte‐mediated antitumor immune response that contributes to cancer progression, which is inconsistent with our results. Notably, a very important factor, namely, RP applied for malignant tumor ablation, is ignored. The above theory is based on the presence of malignant tumors. What changes will occur if the malignant tumor is eliminated via surgery or other techniques? Lorente D analyzed 755 metastatic CRPC patients treated with cabazitaxel or mitoxantrone and found that the conversion from a high pretreatment (≥3) NLR to a low posttreatment (<3) NLR was observed in 43.8% of the patients with high NLR and associated with improved survival (HR 0.66; 95% CI 0.51‐0.85; *P* = 0.001) and higher PSA response rates (66.4% vs 33.6%; *P* = 0.000).^10^ Considering the above evidence, we can reasonably hypothesize that malignant tumors in patients with high NLRs have a stronger ability to stimulate granulopoiesis and suppress lymphopoiesis, with a subsequent elevation in neutrophils and decrease in lymphocytes that promote cancer progression, than malignant tumors in patients with low NLR. If the malignant tumor were to be ablated via surgery or other techniques, the stimulation of neutrophils and suppression of lymphocytes would be relieved, and circulating neutrophils and lymphocytes would be restored back to normal or strengthened to some extent, which also leads to decreased inflammatory carcinogenic factors, strengthened immune surveillance of circulating tumor cells or other minimal malignant lesions and better BCR‐free survival. Regarding patients with low NLRs, the effects of malignant tumors on neutrophils and lymphocytes might be limited, and these tumors might have other mechanisms to avoid immune surveillance. The ablation of malignant tumors slightly affects inflammatory carcinogenic factors and the host immune surveillance of circulating tumor cells or other minimal malignant lesions, which leads to shorter BCR‐free survival. These characteristics of the original malignant tumors might be generalizable to recurrent tumors to a great extent. Once recurrence occurs, the malignant tumors of the patients with high preoperative NLRs will then stimulate granulopoiesis and suppress lymphopoiesis, which leads to increased inflammatory carcinogenic factors, decreased host immune surveillance and poor overall survival. Given that most patients were dismissed within 5 days after surgery in our center and that no plan was made to collect postoperative data from routine blood examinations during follow‐up, it is difficult for the present study to verify the above hypothesis. A well‐designed study is needed to clarify the issue, and we will focus on this topic as a next step.

Several limitations of the present study need to be considered. First, the data for this study were retrospectively collected, and an intrinsic selection bias could not be avoided. The retrospective study design also meant that the postoperative data for routine blood examinations could not be collected, and the hypothesis raised in the paper could not be verified. Second, because all patients in the study were from one center in eastern China, caution should be taken in the generalization of our results to other populations. Prospective multicenter studies are urgently needed. Third, some patients are in the habit of taking antitumor traditional Chinese medicine, and these behaviors, which might have introduced potential bias to the study, were not recorded in our database. Fourth, lymphocyte and neutrophil counts can be influenced by concurrent infections, comorbidities or additional drugs. Although we utilized rigorous exclusion criteria, undetected comorbidities and unreported medication usage, which would also introduce potential bias, may have existed.

## 5 CONCLUSION

The NLR cannot reflect prostate cancer characteristics or the local immune microenvironment, but a high NLR can serve as an independent predictor of better BCR.

## CONFLICT OF INTEREST

None declared.
