# Supplementary material for: The preoperative neutrophil‐to‐lymphocyte ratio is not a marker of prostate cancer characteristics but is an independent predictor of biochemical recurrence in patients receiving radical prostatectomy
Source: Cancer Med. 2019 Jan 28;8(3):1004–12. doi: 10.1002/cam4.1984 (PMC6434220; doi:10.1002/cam4.1984)
Supplement: Supplementary file 1 [file CAM4-8-1004-s001.doc]

Supplemental Table 1 Cox univariate and multivariate analyses of survival according to neutrophil-to-lymphocyte ratio (NLR) in all patients.

|  | BCR-free time | |  | Overall survival time | |
| --- | --- | --- | --- | --- | --- |
|  | HR (95%CI) | P-value |  | HR (95%CI) | P-value |
| Univariate analysis |  |  |  |  |  |
| NLR |  | 0.574 |  |  | 0.040 |
| <2 | 1 |  |  | 1 |  |
| ≥2 | 0.941(0.761-1.164) |  |  | 2.035(1.034-4.004) |  |
| Multivariate analysis |  |  |  |  |  |
| NLR |  | 0.311 |  |  | 0.374 |
| <2 | 1.000 |  |  | 1.000 |  |
| ≥2 | 0.884(0.695-1.123) |  |  | 1.445(0.642-3.254) |  |
| pT stage |  | <0.001 |  |  | 0.214 |
| Low(pT2) | 1 |  |  | 1 |  |
| High(pT3-4) | 2.064(1.588-2.684) |  |  | 0.546(0.211-1.417) |  |
| pN stage |  | <0.001 |  |  | 0.648 |
| Negative | 1 |  |  | 1 |  |
| Positive | 2.355(1.664-3.332) |  |  | 1.426(0.310-6.562) |  |
| Gleason grade |  | <0.001 |  |  | 0.005 |
| Low(<4+4) | 1 |  |  | 1 |  |
| High(≥4+4) | 2.428(1.874-3.145) |  |  | 3.336(1.429-7.789) |  |
| PSA level |  | <0.001 |  |  | 0.794 |
| Low(≤10 ng/ml) | 1.000 |  |  | 1.000 |  |
| High(>10 ng/ml) | 1.904(0.695-1.123) |  |  | 1.128 (0.457-2.787) |  |

(PSA: prostate specific antigen.)
